# Neurocognitive impairment after acute coronary syndrome: Prevalence and characterization in a hospital-based cardiac rehabilitation program sample

**DOI:** 10.15171/jcvtr.2018.11

**Published:** 2018-06-29

**Authors:** Muriela Silva, Eduarda Pereira, Afonso Rocha, Dulce Sousa, Bruno Peixoto

**Affiliations:** ^1^Instituto de Investigação e Formação Avançada em Ciências e Tecnologias da Saúde, CESPU, Gandra, Portugal; ^2^Unidade de Reabilitação Cardíaca/Recondicionamento ao Esforço, Serviço de Medicina Física e de Reabilitação do Centro Hospitalar de São João, Porto, Portugal; ^3^Departamento de Psiquiatria e Saúde Mental do Centro Hospitalar de São João, Porto, Portugal; ^4^Instituto Universitário de Ciências da Saúde, CESPU, Gandra, Portugal; ^5^NeuroGen - Center for Health Technology and Services Research (CINTESIS), Porto, Portugal

**Keywords:** Neuropsychological Assessment, Anxiety, Diastolic Pressure, Cardiovascular

## Abstract

***Introduction:*** Prospective studies have shown the relation between acute coronary syndrome (ACS) and neurocognitive dysfunction with prevalence ranging between 10.51% and 66.8%. The present study aims to determine the prevalence level of neurocognitive dysfunction; the relations between sociodemographic, clinical and emotional variables and neurocognitive functioning in a sample of ACS patients.

***Methods:*** The sample comprised of 53 patients engaged in cardiac rehabilitation within 3 months after an ACS. Patients with any medical history of neuropsychiatric problems prior to the ACS and illiterate subjects were not included in the study.

***Results:*** The majority of the sample (85%) exhibits some degree of cognitive impairment, with 84.8% showing verbal fluency impairment, 60.3% memory impairment and only 26,4% had language compromised. Neurocognitive general functioning was correlated with age. Memory domain was negatively correlated with the number of daily smoked cigarettes before the ACS. Verbal fluency was influenced by schooling. Language domain was correlated with mean diastolic pressure and with the type of profession, visuospatial domain was correlated with schooling, number of cardiovascular risk factors, distress, anxiety levels and type of ACS.

***Conclusion:*** Prevalence rate of neurocognitive dysfunction is considerably high. Besides global neurocognitive functioning, verbal fluency and memory are the most affected domains. Several variables were related to neurocognitive performance: sociodemographic; cardiovascular risk factors; clinical; psychological. The underlying mechanisms of neurocognitive dysfunction should be further explored.

## Introduction

Cardiovascular diseases (CD) are the main cause of mortality and morbidity in Europe.^1,2^ The acute coronary syndrome (ACS) is the most prevalent CD in developing countries.^3^ ACS is the result of the rupture or erosion of the atherosclerotic plaque, with several degrees of thrombosis and distal embolization.^4^

Prospective studies have shown the relation between several CD and lower performance in neurocognitive screening tests.^5^ It is also known that patients with a history of ACS have five-time higher risk of dementia.^6^ Moreover, a cohort study has identified an association between ACS and non-amnesic mild cognitive impairment, mainly characterized by psychomotor, attention and executive functioning alterations.^7^

A six-year follow up study of ACS patients, showed a mild but significant decline of visual memory, visuoconstruction abilities, verbal fluency, executive functioning and global cognitive functioning.^8^ These changes were not related to anesthesia or major surgery.

Deficits in executive functioning, reduction in verbal fluency tasks, psychomotor speed, verbal memory and mental processing speed have been reported.^8-10^ Worse neurocognitive functioning is associated with lower medication adherence and to misunderstand the frequency in which the medication is taken. The prevalence of neurocognitive dysfunction ranges between 10.51% and 66.8%.^10^ The use of diverse neuropsychological instruments and differences in demographic and clinical characteristics (e.g. Treatment, ACS type, comorbidities) of the samples could account for this wide range of prevalence.^10^

Compared to controls, ACS patients present loss of gray matter volume in key areas for high demanding cognitive tasks: left medial frontal cortex, left cingulate and precuneus, left and right parahippocampal gyri and right and left middle temporal gyri.^11^ The executive dysfunction in these patients is associated with an increased functional connectivity in middle-orbito-frontal regions.^12^

The factors that may underlie the neurocognitive alterations in ACS are extensive and far from being completely understood. The severity of the atherosclerosis, ischemia, hypoperfusion, low-grade inflammatory activation, multiple cardiovascular risk factors such as diabetes, central obesity, hypertension, dyslipidemia and psychosocial variables (e.g., anxiety, depression), are some of the variables implicated in this event.^13^

This study aimed to determine the prevalence level of neurocognitive dysfunction; the relations between sociodemographic, clinical and emotional variables and neurocognitive functioning in a sample of ACS patients in a hospital-based cardiac rehabilitation program.

## Materials and Methods

### Participants

A consecutive sample of 53 patients (46 males), engaged in a hospital-based phase II cardiac rehabilitation program (Cardiovascular Rehabilitation Unit, Department of the Physical Medicine and Rehabilitation Service, *Centro Hospitalar de São João*/ Porto, Portugal) after suffering ACS in the last 3 months (Table 1), was included. Participants were completely independent in daily life activities. Several social-demographic and clinical data were extracted from the patient clinical records, including the results on Hospital Anxiety and Depression Scale (HADS) for emotional distress assessment.^14,15^ Professional activity was dichotomized in *white collar* for intellectual/ professional jobs and *blue collar* for manual labors. Patients with any medical history of major systemic or neuropsychiatric problems (e.g., transient ischemic attack, minor and major stroke, dementia) prior to the ACS and illiterate subjects were not included in the study. Every subjected invited has agreed to participate in the study. None were excluded.

**Table 1:** Characteristics of the participants

	**M**	**SD**	**[Min.-Max.]**	**n**	**%**
Age	51.32	10.15	[34-79]		
Schooling (y)	8.87	3.92	[3-16]		
Professional activity					
Blue collar				28	52.8
White collar				25	47.2
Number of CV risk factors	2	1,5	[0-4]		
Previous daily number of cigarettes	13.06	15.17	[0-60]		
Actual daily number of cigarettes	0.68	2.16	[0-10]		
Previous daily alcohol consumption (mg/dL)	21.19	32.24	[0-137.5]		
Actual daily alcohol consumption (mg/dL)	7.68	13.71	[0-72]		
Diagnosis					
With ST segment elevation				35	67.3
Without ST segment elevation				17	32.7
Ventricular ejection fraction (%)	48.77	13.92	[19-70]		
Triglycerides (mg/dL)	148.4	73.34	[47-374]		
Cholesterol (mg/dL)					
HDL	39.23	7.92	[28-57]		
LDL	119.12	39.89	[53-224]		
Glucose (mg/dL)	95.51	27.14	[70-216]		
Mean blood pressure (mm Hg)					
Systolic	116.83	15.98	[90-153]		
Diastolic	70.09	10.35	[60-93]		
BMI (kg/m^2^)	27.9	4.08	[20-41]		
Waist circumference (cm)	96.4	11.56	[68-127]		
HADS (Total)	12.28	9.04	[0-33]		
HADS (Depression)	5.31	4.6	[0-16]		
HADS (Anxiety)	7.26	4.79	[0-18]		
Medication					
Antiplatelet				53	100
Statins				53	100
Angiotensin inhibitors				49	92.5
Betablockers				47	88.7
Nitroglycerine (SOS)				42	79.3
Anxiolytics				22	41.5
Diuretics				11	20.8
Antidiabetics				11	20.8
Other				53	100

### Neurocognitive assessment

All participants were administered the Portuguese version of the Addenbrooke’s Cognitive Examination-III (ACE-III) for neurocognitive assessment.

ACE-III is a neurocognitive screening test, evaluating different cognitive dimensions and enabling an overall picture of the subject’s neurocognitive functioning.

ACE-III is scored out of 100 and assesses five cognitive domains: Attention (maximum score 18 points); Memory (maximum score 26 points); Verbal fluency (maximum score 14 points); Language (maximum score 26 points); Visuospatial (maximum score 16 points). With the sum of the domains, a global indicator of cognitive functioning is obtained.^16^ ACE-III has normative data for the Portuguese population, therefore allowing the conversion of raw scores into *z* scores, according to age and schooling of the participants. The conversion formulas and administration norms are published elsewhere.^17^

Once *z* scores are obtained, participants performance could be classified as follows: *z* score ≥-1, Normal; z score <-1 and ≥-1.5, Mild Deficit; z score < -1.5 and ≥-2, Significant Deficit; z score <-2, Severe Deficit.

### Procedure

Participants were selected at the time of their first appointment at the cardiac rehabilitation program. Immediately after the scheduled appointment, the neurocognitive assessment was performed in one session.

### Statistical analysis

Statistical analysis was carried out using the IBM Statistics version 23 for Windows software.

Measures of central tendency and frequency were used to describe the obtained results and to define the prevalence of neurocognitive dysfunction in the sample.

The study of the relations between continuous variables was determined by Pearson’s correlations. The relation between nominal and continuous variables was studied through the Mann-Whitney U test and Kruskal-Wallis test. Results with *P* ≤ 0.05 were considered significant.

## Results

The results obtained on ACE-III and its domains are shown in Table 2.

**Table 2:** Results obtained on ACE-III and its domains expressed in z scores

** **	**M**	**SD**	**[Min.-Max]**
**ACE-III**	-1.97	1	[-6.13- 0.69]
Attention	-0.52	1.02	[-2.97- 1.18]
Memory	-1.72	1.8	[-5.59- 1.42]
Verbal fluency	-2.4	1.16	[-5.8- -0.11]
Language	-0.68	1.35	[-5.47-1.7]
Visuospatial	-0.87	1.58	[-8.67-1.34]

Table 3 shows the classification of the sample’s performance on ACE-III and its domains. The majority of the sample (85%) exhibits some degree of cognitive impairment, 49.1% present severe impairment. Verbal fluency and memory are the cognitive domains that contribute the most to the negative level of neurocognitive functioning. 84.8% of the participants have a verbal fluency impairment, 50.9% in the severe form. Memory follows verbal fluency with 60.3% of the participants impaired, 50.9% present severe impairment. Language is the less affected domain with 73.6% of the participants showing no impairment.

**Table 3:** Classification of the performance on the neurocognitive assessment

		**n**	**%**
ACE-III			
	No deficit	8	15.1
	Mild deficit	8	15.1
	Significant deficit	11	20.8
	Severe deficit	26	49.1
Attention			
	No deficit	37	69.8
	Mild deficit	7	13.2
	Significant deficit	2	3.8
	Severe deficit	7	13.2
Memory			
	No deficit	21	39.6
	Mild deficit	1	1.9
	Significant deficit	4	7.5
	Severe deficit	27	50.9
Verbal fluency			
	No deficit	8	15.1
	Mild deficit	5	9.4
	Significant deficit	13	24.5
	Severe deficit	27	50.9
Language			
	No deficit	39	73.6
	Mild deficit	2	9.4
	Significant deficit	5	3.8
	Severe deficit	7	13.2
Visuospatial			
	No deficit	32	60.4
	Mild deficit	5	9.4
	Significant deficit	6	11.3
	Severe deficit	10	18.9

ACE-III results showed a negative correlation with age (ρ=-.278; *P* = 0.044). Memory domain was negatively correlated with the number of daily smoked cigarettes before the ACS (ρ=-.291; *P* = 0.035). Verbal fluency was solely influenced by schooling (ρ=.299; *P* = 0.029). Language domain was positively correlated with mean diastolic pressure (ρ=-.299; *P* = 0.006) and participants with a *white collar* (Mean rank= 29.08) profession obtained higher results (U=298; *P* = 0.023) on this domain when compared to those with a *blue collar* occupation (Mean rank= 25.14). Visuospatial domain was correlated with schooling (ρ=-.571; *P* < 0.001), number of cardiovascular risk factors (ρ=-.426; *P* = 0.001), HADS total score (ρ=-.388; *P* = 0.01) and HADS anxiety component (ρ=-.416; *P* = 0.006). Participants with the diagnosis of ACS (U= 372; *P* = 0.04) without ST segment elevation (Mean rank = 30.88) obtained higher results on visuospatial domain when compared to those with ST segment elevation (Mean rank = 24.37).

## Discussion

The present study has determined a prevalence of 85% of neurocognitive impairment in a sample of ACS patients at the beginning of a hospital-based cardiac rehabilitation program. It is known that ACS patients tend to exhibit lower performance on neurocognitive screen measures ^11,18,19^ even when compared to transient ischemic attack and minor stroke patients.^20^ Even in animal models of myocardial ischemia, the triggering of neurocognitive dysfunction is notorious.^21^ However, our prevalence value is higher than those previously reported.^10^ This may be due to the fact that we have used a hospital sample three months after the ACS. Interestingly, a prospective study has established a neurocognitive assessment at 3 months after ACS as a baseline.^8^ Despite the percentage of impairment based on tests *z* scores not being revealed, the authors state that there were a number of participants in the coronary groups who had baseline scores more than 2SD below the median.^8^

Verbal fluency and memory were the most impaired domains. Verbal fluency refers to non-motor processing speed, language generation and executive functioning. It relies on the left dorsolateral prefrontal cortex and temporal lobes.^22^ Executive function impairments are frequently reported in coronary patients,^9,10^ associated in particular with verbal fluency and general cognitive function impairments.^23^ These alterations are related to an increased functional connectivity in prefrontal regions.^12^ Memory, especially verbal memory,^20^ is another domain frequently implied in coronary disease. A recent animal study showed that after myocardial ischemia, reperfusion leads to reactive gliosis in hippocampal subregions CA1, CA3 and dentate gyrus, thus suggesting an inflammatory basis for the memory deficits.^21^

General cognitive functioning, measured through ACE-III total score, was negatively influenced by age. It is well known that age influences cognitive functioning, however, the ACE-III results were already converted (*z* scores) according to age and schooling. Thus, this result clearly points to an augmented impact of higher age on cognition after ACS. Research has pointed to the role of cardiovascular risk factors as etiological factors in cognitive decline in ageing.^24^ Perhaps the enduring of cardiovascular risk factors in association with higher age could account for this relation.

Memory functioning was influenced by the number of daily smoked cigarettes prior to the coronary event. Smoking is one of the most known modifiable cardiovascular risk factors. Even in the absence of cardiovascular disease, smoking is associated with extensive annual change in white matter hyperintensity volume, total brain volume and temporal horn volume, which is a marker of hippocampal atrophy.^25^

Verbal fluency was correlated with schooling, a well-known factor of cognitive reserve,^26^ which has the same effect on visuospatial domain.

Language showed a positive correlation with diastolic pressure. The relation between blood pressure and poor cognition is U-shaped, since low diastolic pressure is associated with reduced cerebral perfusion.^27^ However, it is not clear the reason for this relation with language domain. Language performance was also associated with the nature of the profession of participants, reflecting a clear practice of *white collar* occupations.

Visuospatial domain was influenced, besides schooling, by the number of cardiovascular risk factors, emotional distress (HADS total score), anxiety (HADS anxiety) and the diagnosis of ACS with ST segment elevation. The cumulative effect of cardiovascular risk factors has been pointed out, concerning global cognitive functioning.^20^ Emotional distress in the form of anxiety is associated with poor health behaviors and therefore to the accumulation of cardiovascular risk factors ^28^. It has been proposed that in addition to behavioral factors, the sympathetic hyperactivity in response to distress can lead to left ventricular dysfunction, decreased baroreflex dysfunction and decreased heart rate variability.^28,29^

The present study has some limitations. The reduced number of participants and the narrowed inclusion criteria, limits the extent of the conclusions. Nevertheless, since the sample is initiating a cardiac rehabilitation program, in the near future the impact of the program on neurocognition can be assessed. The neurocognitive assessment was based on a neuropsychological screening test. Ideally, the assessment should have been comprehensive. The use of an extensive battery of tests should provide more information about neurocognitive functioning, however, it would be extremely time-consuming and therefore inadequate on the clinical context of this study.

It would be interesting to study the neurovascular involvement of these patients by arterial spin-labeling MRI, since it has been proposed a vascular basis for neurocognitive involvement.^13^ The association between ACS and the development of dementia also fosters the idea of a vascular basis for neurodegeneration.^6,30^

## Conclusion

The prevalence rate of neurocognitive dysfunction in ACS patients is very high. Besides global neurocognitive functioning, verbal fluency and memory are the most affected domains.

Several variables were related to neurocognitive performance: sociodemographic (age, schooling and type of profession); cardiovascular risk factors (number of daily cigarettes prior to ACS and the number of risk factors); clinical (diastolic pressure and type of ACS); psychological (emotional distress and anxiety).

Neuropsychological assessment should be mandatory for these patients in order to identify neurocognitive deficits and to implement neuropsychological rehabilitation programs aiming to reduce the impact of the disease on cognition.

The underlying mechanisms of neurocognitive dysfunction should be further explored.

## Ethical approval

This study was approved by the ethics committee of the Centro Hospitalar de São João, EPE. All participants gave their informed consent.

## Competing interests

All authors declare no competing financial interests exist.
